# Non-Migraine Head Pain and Botulinum Toxin

**DOI:** 10.3390/toxins16100431

**Published:** 2024-10-09

**Authors:** Fatemeh Farham, Dilara Onan, Paolo Martelletti

**Affiliations:** 1 Department of Headache, Iranian Centre of Neurological Research, Neuroscience Institute, Tehran University of Medical Sciences, Tehran 1417653761, Iran; 2 Department of Physiotherapy and Rehabilitation, Faculty of Heath Sciences, Yozgat Bozok University, Yozgat 66000, Turkey; dilara.onan@yobu.edu.tr; 3 School of Health, Unitelma Sapienza University of Rome, 00161 Rome, Italy

**Keywords:** botulinum toxin-A, trigeminal neuralgia, tension-type headache, cluster headache, post-traumatic headache, cervicogenic headache

## Abstract

Botulinum toxin A (BT-A), a potential neurotoxin produced by the bacterium Clostridium botulinum, is known for its ability to prevent the release of acetylcholine at the neuromuscular synapse, leading to temporary muscle paralysis. BT-A is used for a wide range of therapeutic applications. Several studies have shown mechanisms beyond the inhibition of acetylcholine release for pain control. BT-A inhibits the release of neurotransmitters associated with pain and inflammation, such as glutamate, CGRP, and substance P. Additionally, it would be effective in nerve entrapment leading to neuronal hypersensitivity, which is known as a new pathogenesis of painful conditions. BT-A has been applied to the treatment of a wide variety of neurological disorders. Since 2010, BT-A application has been approved and widely used as a chronic migraine prophylaxis. Moreover, due to its effects on pain through sensory modulation, it may also be effective for other headaches. Several studies using BT-A, at different doses and administration sites for headaches, have shown beneficial effects on frequency and severity. In this review, we provide an overview of using BT-A to treat primary and secondary headache disorders.

## 1. Introduction

Globally, headache disorders affect approximately 40% of the population. For most age groups, they rank among the three most common neurological diseases [1]. The ICHD-3 classifies more than 150 different types of headaches, which are caused by various factors such as medications, symptoms of other diseases, environmental factors, and other causes [2]. In 2019, the latest Global Burden of Disease (GBD) study estimated that a total of 46.6 million years of healthy life lost due to disability (YLD) were attributed to headaches [3]. Headache disorders are among the most common and disabling conditions; therefore, services and care pathways should be available to all age groups and patients with varying conditions [4,5].

Treatment includes acute pain control followed by preventive treatment. Frequent use of painkillers may be associated with a type of headache, called medication overuse headache, which can increase pain in the long term instead of reducing it. Additionally, there are limitations in medication due to side effects [6]. Migraine is a very common disorder; as a chronic disease, it ranks as the third most common and the seventh most debilitating disease worldwide among people under 50 years of age [7]. In the 1990s, it was discovered that patients experienced a reduction in migraine headaches after receiving injections of onabotulinumtoxinA for hyperfunctional facial lines [8].

Since 2010, Botulinum toxin-A (BT-A) injections have been approved as a chronic migraine prophylaxis based on placebo-controlled trials (PREEMPT 1 and PREEMPT 2) [9,10]. Phase III trials showed a significant reduction in headache days, frequency of attacks, and use of abortive treatment, along with a positive effect on quality of life and good safety [10]. Currently, BT-A is approved only for the treatment of chronic migraine, but it may also be effective for other headaches [8,11]. In this article, we review the effects of BT-A on primary and secondary headaches other than migraine based on clinical studies.

## 2. Botulinum Toxin-A

Clostridium botulinum can produce seven immunologically distinct botulinum neurotoxins (types A through G) that cause botulism paralysis. Toxin types A to F are involved in human botulism, animal botulism, or both, while Type G was isolated from soil samples [12]. Serotype A is the only commercially available botulinum toxin for clinical use. Three known formulations of botulinum toxin A exist: Dysport, Botox, and Xeomin. Differences in these toxins may relate to variations in the strain of bacterium, preparation method, diffusion, and potency testing [13]. Studies have shown that a unit of Botox is three times as potent as a unit of Dysport [14].

The potent neurotoxin BT-A is produced by the bacterium Clostridium botulinum, preventing acetylcholine release at the neuromuscular synapse, leading to muscle paralysis. While this mechanism is wellknown, it does not fully explain the analgesic effect of BT-A on painful conditions such as migraine, trigeminal neuralgia, and other neuropathic pain [15]. Several preclinical studies have shown that BT-A inhibits the release of neurotransmitters associated with pain and inflammation [16,17,18], including local pain neuropeptides like glutamate, substance P, and CGRP [19,20,21]. Furthermore, BT-A has been demonstrated to affect the transient receptor potential vanilloid 1 (TRPV1), responsible for processing thermal and noxious stimuli. It reduces the delivery of this receptor to cell membranes and can diminish peripheral sensitization, neurogenic inflammation, and pain [22,23].

Recent research has explored a new paradigm in headache pathogenesis, focusing on the entrapment and compression of sensory nerves in the head and neck region, which can trigger a local neuroinflammatory response by releasing various neuropeptides. This process can lead to neuronal hypersensitization, manifesting clinically as hyperalgesia and allodynia [24]. These factors can cause peripheral and central sensitization ultimately resulting in chronic pain. BT-A could be an effective treatment for these etiologies of headache disorders by blocking the release of these neurotransmitters [24].

### Research Overview on Non-Migraine Head Pain and Botulinum Toxin

The use of botulinum toxin A (BT-A) for managing non-migraine head pain has garnered significant attention in the scientific community. We reviewed human studies on the effect of BT-A on headaches. We used articles including clinical trials, case series, and case reports. The following summary highlights key findings from publications, categorized by their focus areas.

## 3. Trigeminal Neuralgia

Trigeminal neuralgia (TN) is a severe, episodic, unilateral facial pain that patients typically experience as brief, stabbing attacks triggered by eating, drinking, talking, and touching the face [25]. The first step in treatment is medication such as Carbamazepine. However, these medications are poorly tolerated due to multiple daily doses, side effects, and inadequate efficacy over time [26,27]. In addition to non-invasive options, minimally invasive procedures are destructive, and microvascular decompression surgery, which preserves the trigeminal nerve, may have serious complications, such as aseptic meningitis, cerebrospinal fluid leaks, and ipsilateral hearing loss [28].

Despite recent advances in its treatment, the management of TN remains a significant challenge. After the relief of TN pain in a patient who underwent BT-A injection due to hemifacial spasm [29], the BT-A injection’s effect on TN was investigated in studies. BT-A can be effective in treating TN through its analgesic effects.

Several clinical trials and open-label studies showed the effect of BT-A on the treatment of TN with several indicators such as the percentage of recovery, reduction in attack frequency, and reduction in pain intensity measured through visual analog scale (VAS) and numeric pain rating scale (NSR) criteria [30,31,32,33,34,35,36,37,38,39,40,41]. In addition, one study showed that BT-A can also reduce the dose or number of preventive medications (including Carbamazepine and Oxcarbamazepine) or discontinue them [42].

In these studies [30,31,32,33,34,35,36,37,38,39,40,41], 25–200 international units were injected. The effects of injecting 25 units [35], 50 units [40], and 75 units [35,41] have been significant in randomized, double-blind, placebo-controlled trials. In a study that compared the effect of 25 units with 75 units, it was shown that both had similar effectiveness in the short term [35].

There were no serious adverse events in all these studies. All the side effects were mild or moderate and transient and did not require further management. Tenderness, erythema, edema, hematoma, itching, and pain were reported at the injection site, which resolved within 2 to 7 days. Transient facial asymmetry in the injection area was reported, which disappeared within 6 to 7 weeks [35,37,41]. In a study, transient paresis of the buccal branch of the facial nerve was reported in three patients, one of whom had severe paresis and needed physical therapy, and paresis disappeared after 3 months [39]. The results of these articles demonstrate that BT-A is a less invasive and effective option that can be used as a first-line treatment before other non-pharmacological and invasive methods [31,32,34,35,36,37,38,43].

## 4. Tension-Type Headache

Tension-type headache (TTH) is a primary headache characterized by head-pressing and bilateral pain that wraps around the head like a band and is associated with tender pericranial muscle tissue [2]. TTH is the most common type of primary headaches, and a study conducted in 2021 estimated the 1-year prevalence in the population to be 26.8% [44]. TTH creates burdens for individuals due to the economic costs of treatment, loss of working days, and disruption of family relationships, and these burdens increase as the condition becomes chronic [45,46]. Disability, workday losses, and increased treatment costs in TTH can affect individuals’ quality of life across nearly every aspect of their lives [47,48].

NSAIDs and acetaminophen are effective pharmacological treatments for TTH [46,49,50]. However, they are not a disease-specific pharmacological approach for acute headaches [49]. Limited studies indicate that triptans are not recommended in TTH [46,51,52]. Again, pharmacological preventive treatment is not specific to the disease [46], but it is stated that amitriptyline, mirtazapine, and venlafaxine are effective on chronic TTH (CTTH) [45,46]. However, the pathophysiology of TTH is still not clear, and there are points where it overlaps with migraine. In addition, considering the risk of medication overuse in headaches associated with pharmacological treatment, various non-pharmacological approaches are being investigated [46].

BT-A may be considered as an option in the prophylactic treatment of TTH [53]. The thought mechanism of BT-A in TTH is the reduction in pericranial muscle tension by cholinergic chemodenervation [54], inhibition of calcium-dependent CGRP from trigeminal neurons, and inhibition of pain mediators such as substance P, bradykinin, and glutamate by degradation of SNAP-25 [55,56].

Limited randomized controlled trials and case studies in the literature indicate that BT-A reduces headache intensity and frequency in CTTH, while a systematic review and meta-analysis found no significant benefit of BT-A on headache symptoms in CTTH [57]. A recently published systematic review and meta-analysis examined the results of 11 randomized controlled studies. BT-A has been shown to provide significant benefits in headache frequency, duration, and acute pain medication use in CTTH patients compared to controls; however, it has been reported that studies in the literature are limited in terms of the level of evidence [48]. The mean (range) value of the doses used in these studies was stated to be about 178 (20–500) units [48]. However, it was reported that the optimal dose for ideal treatment protocols is still unclear, and the optimal dose, to be determined according to age or gender, should be targeted in future studies.

## 5. Trigeminal Autonomic Cephalgias (TACs)

TACs are characterized by unilateral headaches associated with prominent cranial parasympathetic autonomic features, ipsilateral to the headache. Headaches in this group are mainly classified according to the duration of symptoms [2].

### 5.1. Cluster Headache

Cluster headache (CH) is the most common type of TAC characterized by very severe, unilateral pain, around the orbit, supraorbital, and temporal regions, with autonomic symptoms on the side of pain that lasts 15–180 min [2]. The lifetime prevalence is greater than 1 in 1000 [58]. These severe attacks significantly impact the performance and quality of life of patients. Acute treatment includes oxygen therapy and triptans [59,60]. One of the effective prophylactic treatments for CH is verapamil [61]. In clinical practice, it has been shown that there is a need to use relatively higher doses of verapamil for CH, even higher than the doses used in cardiac indications [62,63]. Verapamil’s side effects such as bradycardia, ankle edema, and constipation can limit the use of this medication at proper dosage [64]. Chronic CH (CCH) is characterized by no remission or periods of remission less than 3 months and may be resistant to medical treatment [65].

The effectiveness of botulinum toxin in cluster headaches has been investigated in several studies [66,67,68,69,70]. Two studies evaluated the effect of BTA injection on ganglions [66,70]; Otic ganglion was not a good target for CH treatment [70]; however, another study reported that 25 or 50 IU injections of BT-A around the SPG reduced the number of cluster headache attacks in a statistically significant manner in an intention-to-treat analysis [66].

BT-A injection was investigated in two other studies. In one of them, a cumulative dose of 50 international units (IU) of BT-A was injected into the temporalis (10 IU), frontalis (10 IU), splenium capitis (10 IU), and trapezius (20 IU) muscle ipsilateral to the pain. In the other study, the injection was performed based on the PREEMPT study protocol. In both studies, significant reductions in headache frequency, pain intensity, and mean headache minutes of chronic CH (CCH) were observed. In the two latest articles, BT-A was well tolerated, and in a few patients, mild and transient adverse events were reported, such as eyebrow ptosis, worsening of headache before improvement, and mild neck muscle weakness [67,68]. Botulinum toxin could be an effective adjunctive treatment for CCH; however, the usefulness of BT-A as a therapeutic option needs to be confirmed in double-blind, randomized, controlled studies.

### 5.2. Hemicrania Continua

Hemicrania continua (HC) is a continuous unilateral headache, typically mild to moderate in intensity, with severe exacerbations accompanied by cranial autonomic features. It responds strongly to indomethacin [2]. However, some patients cannot tolerate this treatment, due to gastrointestinal complications [71]. Two case reports and one prospective study examined the effect of BT-A on HC [72,73,74]. Sara Miller and colleagues conducted a study on nine patients with refractory HC who received BT-A according to the PREEMPT protocol. Their results showed that five patients experienced more than a 50% reduction in moderate or severe headache days, along with significant improvements in both HIT-6 and MIDAS scores. Some adverse effects such as transient eyebrow ptosis, frontalis overactivity, and transient worsening in headache before improvement were reported in three subjects [74]. BT-A may be a potential alternative for patients who are refractory or intolerant to indomethacin therapy. However, more controlled studies are needed to clarify the efficacy of BT-A in treating HC.

### 5.3. Short-Lasting Unilateral Neuralgiform Headache with Conjunctival Injection and Tearing (SUNCT)

SUNCT is a rare type of TAC, mainly characterized by severe, very frequent, unilateral attacks of pain in the ocular and periocular regions, accompanied by conjunctival injection and tearing, lasting 1–600 s [2]. Medication is the first line of treatment. Lamotrigine, gabapentin, topiramate, and carbamazepine have been successfully used in a cohort of patients. However, the treatment of SUNCT is challenging, and no randomized controlled trials have been conducted [75].

Two case reports showed the efficacy of BT-A injection in refractory SUNCT patients, both of whom became pain-free without any side effects [76,77]. In one case, 70 IU of BT-A was injected around the orbit, temporal region, and upper gum of a 12-year-old boy. The frequency and severity of his pain decreased by the 4th day after treatment, and he was pain-free after 7 days [76]. In another case, Ramon J Zabalza reported a 56-year-old man who dramatically responded to BT-A injection in four points around the orbit [77]. BT-A may represent a new therapeutic approach for refractory cases of SUNCT and warrants further investigation in studies.

## 6. Cervicogenic Headache

Cervicogenic headache (CeH) is typically characterized by unilateral pain in the back of the head and neck, and it is a secondary headache caused by dysfunction of cervical spine structures such as vertebrae, discs, and soft tissues [2,78]. Decreased joint range of motion in the neck and dysfunction of the deep flexor muscles may also be observed [79]. The literature suggests that, these disorders can be treated long-term with manual therapy and motor control exercises [79]. A multidisciplinary approach is considered important, as pharmacological treatments are often ineffective, prompting the search for alternative therapies [79]. BT-A has been applied in limited studies, with satisfactory results for treatment of chronic migraine, and other headaches, targeting acetylcholine and pain mediators. In a single study, significant improvements in pain intensity and cervical joint range of motion were reported, 2 and 4 weeks after the application [80]. In the study of Karadaş et al., headache intensity and frequency decreased significantly at 6 and 12 weeks after treatment compared to before treatment [81]. The reported side effects included localized pain at the injection site and neck muscle weakness [81]. Schnider et al. reported significant improvements in headache intensity, frequency, and neck muscle sensitivity at the end of the 12th week of their study, which compared physical therapy combined with BT-A treatment to physical therapy combined with placebo BT-A [82]. However, in a crossover study by Linde et al., no improvement was observed in headache intensity, frequency, neck pain severity, analgesic use, quality of life, cervical joint range of motion, and pressure pain threshold 8 weeks after the application [83]. According to the limited studies in the literature, the exact mechanism of pain in CeH is not fully understood. The uncertainty about whether the muscle is hyperactive or if a different myofascial condition is influencing it may cause insufficient results [84]. Roland et al. [78], in their systematic review and meta-analysis, reported that they could not confirm the prophylactic effect of BT-A treatment in CeH. They noted that the studies used varying doses of 90 IU [82], 100 IU [80,83], or 150 IU [81] and there was no standardized dosage. The reasons for not being able to confirm the prophylactic effect may be the differences in dose and duration of treatment and the small number of studies. Therefore, they emphasized the need for high-quality randomized controlled trials [84].

## 7. Post-Traumatic Headache

According to ICHD-3, post-traumatic headache (PTH) is defined as a headache that begins within 7 days and lasts for more than 3 months after mild, moderate, or severe traumatic brain injury [2]. It is noteworthy that BT-A application in PTH, as with other secondary headaches, is rarely mentioned in the literature. It has been noted that the pain may resemble characteristics of migraine and tension-type headaches [85]. Therefore, similar treatments used for these headache types can be applied to PTH [85]. Soldiers who sustain traumatic brain injuries are at an increased risk for PTH. In a study by Yerry et al., 64 soldiers were treated with BT-A (155 to 200 units), and 41 (64%) showed improvement [86]. In a survey of veterans in Los Angeles, weekly headache days and headache intensity significantly improved from baseline to the 12th week after the application of 387.5 units of aboBT-A. The side effects were transient and mild, including pain, forehead paresthesia, itching, dizziness, and ocular symptoms [85]. In three case studies of chronic PTH following gunshot wounds, BT-A treatment led to a 70–100% improvement in headache and neck pain severity, as well as muscle spasms, 4 weeks after application [87]. These studies targeted pain mediators in sensory neurons as a potential mechanism by which BT-A provides relief. However, the limited number of studies available may not fully represent the broader patient population. Since the underlying pathology in post-traumatic headache is trauma, it may be difficult to isolate the specific effect of BT-A [88], and further high-quality studies are needed.

## 8. Discussion

In this review, we provide evidence for the effect of BT-A on primary and secondary headaches. Clinical studies have demonstrated the analgesic effect of BT-A and its role in the treatment of chronic pain, including headache disorders [15]. Headache is one of the most common neurological disorders, significantly impacting the quality of life and increasing the years of life with disability [1,3]. According to ICHD-3, headaches are categorized as primary or secondary based on etiology and as episodic or chronic based on attack frequency [2]. BT-A injections were first suggested as an effective headache treatment when it was observed that individuals suffering from chronic headaches experienced improvement after receiving cosmetic BT-A injections [89,90,91]. Following this, numerous studies were conducted on the effect of BT-A on headaches, culminating in FDA approval for the treatment of chronic migraine in 2010 [92]. Early studies showed that BT-A inhibits the release of acetylcholine from the motor and autonomic nerve terminals. Later research, aimed at understanding its mechanisms in chronic migraine, overactive bladder, and depression, revealed findings such as the role of SNARE complexes in the delivery of TRPV1 to membranes and the role of afferent feedback [8]. According to the PREEMPT injection paradigm, a standardized protocol has been established for chronic migraine. Patients received intramuscular injections of onabotulinumtoxinA at 31 fixed injection sites and a fixed dose in the head and neck muscles. The PREEMPT studies found marked improvement in several headache symptoms and demonstrated that treatment was associated with improved patient functioning, vitality, and overall quality of life, as well as reduced psychological distress [9,10,91,92,93]. Monoclonal antibodies targeting CGRP have been developed specifically for the prophylactic treatment of chronic migraine. In an indirect comparison of CGRP monoclonal antibody and BT-A in the treatment of chronic migraine, Jiajie Lu et al. observed no significant difference in the changes in migraine days, headache days, HIT-6 score, and 50% migraine responder rate between CGRP monoclonal antibody and botulinum toxin [94].

Tension-type headache is the most common type of headache [44]. It imposes a significant economic burden and disruption of family relationships, with the burden increasing as the condition becomes chronic [45,46]. William G. Ondo and colleagues investigated the efficacy of BT-A in treating chronic daily headaches, including TTH and migraine, in a randomized, double-blind, placebo-controlled trial. There were 40 participants in each group, with each group containing 23 chronic migraine patients and 7 patients with CTTH. They administered 200 units of BT-A using a “follow the pain” strategy to select injection sites. The number of headache days in the BT-A group decreased significantly between weeks 8 and 12 after initial treatment [95]. Recently, a systematic review and meta-analysis of 11 randomized controlled trials has shown that BT-A provides significant benefits in reducing headache frequency, duration, and acute pain medication use in CTTH patients compared to controls [48]. However, more trials with a higher level of evidence are needed. TN is characterized by severe, episodic, unilateral facial pain [25]. Due to the need for multiple daily doses, side effects, and inadequate long-term efficacy, medications for TN are often poorly tolerated, and other non-medical procedures can be invasive with serious complications [26,27,28]. There are several case series and clinical trials that have investigated the effect of BT-A on TN, all of which demonstrated significant benefits in attack severity and frequency [30,31,32,33,34,35,36,37,38,39,40,41]. However, more double-blind randomized controlled trials with larger sample sizes should be conducted. The effectiveness of botulinum toxin in cluster headaches has also been investigated, showing that BT-A injections significantly reduced headache frequency, pain intensity, and mean headache minutes in CCH [66,67,68,69,70]. BT-A could be a useful adjunctive treatment for CCH; however, there are currently no double-blind, randomized, controlled studies available. Similarly, there are no randomized controlled trials for other types of TACs. However, case reports and prospective studies have indicated that BT-A is effective in HC [71,72,73]. Given the poor tolerance of indomethacin [70], BT-A can be considered as a preventive treatment for HC. Some studies have suggested the effectiveness of BT-A in cervicogenic headache, but meta analysis have not confirmed its role in headache prevention [79,80,81,83]. The exact mechanism of cervicogenic headache is not well understood [83], which may lead to inconsistent results and highlights the need for further investigation. Post-traumatic headache is another secondary headache that mimics migraine and TTH, allowing for similar treatment strategies. In one study, veterans showed a significant beneficial effect of BT-A; however, more studies are needed to confirm the efficacy of BT-A in treating post-traumatic headache [85]. Besides preventive treatments, acute treatments should also be provided for headaches. Ubrogepant is a CGRP receptor antagonist that may have a potential synergistic effect when combined with BT-A injection [96]. BT-A is a well-tolerated treatment option. Adverse effects such as localized pain, tenderness, and bruising at the injection site usually occur within the first two weeks after injection. Muscle weakness may occur if the toxin spreads, but hypersensitivity reactions are rare [97]. Anxiety and depression are more prevalent in individuals with primary headaches and show a positive correlation with the frequency and intensity of headaches [46]. Several studies have indicated that when BT-A is used to treat glabellar frown lines, spasticity, dystonia, and chronic migraine, comorbid anxiety disorders or related symptoms improve, supporting the hypothesis that BT-A may have an anxiolytic effect [98,99,100,101,102].

Despite these findings, double-blind case-control studies are needed to confirm BT-A as a preventive treatment for different headache types. Important limitations of this review are the insufficient number of controlled trials and the small sample size in each study.

## 9. Conclusions

The reviewed studies demonstrate that botulinum toxin type A (BT-A) could be a logical option in the treatment of various types of head pain, particularly those caused by nerve entrapment and chronic conditions. However, more clinical trials are needed to accept and validate BT-A as an effective treatment. The total number of results retrieved indicates a robust interest and diverse research focus on this therapeutic approach.

## 10. Future Directions

Considering the positive effects of BT-A on various headaches, further investigation is needed through randomized controlled trials with larger sample sizes. For each type of headache, it is important to establish a specific protocol that determines the required dose and injection points based on the mechanism and location of the pain.

## Data Availability

Not applicable. No new data have been created.
